# Fluid removal associates with better outcomes in critically ill patients receiving continuous renal replacement therapy: a cohort study

**DOI:** 10.1186/s13054-020-02986-4

**Published:** 2020-06-01

**Authors:** Anna Hall, Siobhan Crichton, Alison Dixon, Ilia Skorniakov, John A. Kellum, Marlies Ostermann

**Affiliations:** 1 Department of Critical Care, Guy’s & St Thomas’ Hospital, London, UK; 2 Medical Research Council Clinical Trials Unit, University College London, London, UK; 3 Department of Nephrology and Dialysis, Sverdlovsk Regional Clinical Hospital 1, Yekaterinburg, Russia; 4 Department of Critical Care Medicine, Center for Critical Care Nephrology, University of Pittsburgh, Pittsburgh, PA USA; 5 Department of Critical Care, King’s College London, Guy’s & St Thomas’ Hospital, Westminster Bridge Road, London, SE1 7EH UK

**Keywords:** Renal replacement therapy, Acute kidney injury, Fluid balance, Fluid management, Ultrafiltration, Fluid removal

## Abstract

**Background:**

Fluid overload is associated with morbidity and mortality in patients receiving renal replacement therapy (RRT). We aimed to explore whether fluid overload at initiation of RRT was independently associated with mortality and whether changes in cumulative fluid balance during RRT were associated with outcome.

**Methods:**

We retrospectively analysed the data of patients who were admitted to the multidisciplinary adult intensive care unit (ICU) in a tertiary care centre in the UK between 2012 and 2015 and received continuous RRT (CRRT) for acute kidney injury for at least 24 h. We collected baseline demographics, body mass index (BMI), comorbidities, severity of illness, laboratory parameters at CRRT initiation, daily cumulative fluid balance (FB), daily prescribed FB target, fluid bolus and diuretic administration and outcomes. The day of the lowest cumulative FB during CRRT was identified as nadir FB.

**Results:**

Eight hundred twenty patients were analysed (median age 65 years; 49% female). At CRRT initiation, the median cumulative FB was + 1772 ml; 89 patients (10.9%) had a cumulative FB > 10% body weight (BW). Hospital survivors had a significantly lower cumulative FB at CRRT initiation compared to patients who died (1495 versus 2184 ml; *p* < 0.001). In the 7 days after CRRT initiation, hospital survivors had a significant decline in cumulative FB (mean decrease 473 ml per day, *p* < 0.001) whilst there was no significant change in cumulative FB in non-survivors (mean decrease 112 ml per day, *p* = 0.188). Higher severity of illness at CRRT initiation, shorter duration of CRRT, the number of days without a prescribed FB target and need for higher doses of noradrenaline were independent risk factors for not reaching a FB nadir during CRRT. Multivariable analysis showed that older age, lower BMI, higher severity of illness, need for higher doses of noradrenaline and smaller reductions in cumulative FB during CRRT were independent risk factors for ICU and hospital mortality. Cumulative FB at CRRT initiation was not independently associated with mortality.

**Conclusion:**

In adult patients receiving CRRT, a decrease in cumulative FB was independently associated with lower mortality. Fluid overload and need for vasopressor support at CRRT initiation were not independently associated with mortality after correction for severity of illness.

## Introduction

In critically ill patients, haemodynamic stabilisation often involves aggressive fluid resuscitation and vasopressor support. Although considered to be crucial for optimisation, this approach tends to lead to fluid accumulation. Evidence is accumulating that fluid overload is associated with harm, including worsening organ dysfunction and mortality [1–8]. Patients with acute kidney injury (AKI) are particularly at risk. For instance, in patients with early AKI, fluid overload increases the risk of worsening AKI [9]. In patients treated with renal replacement therapy (RRT), fluid overload on the first day of RRT correlates with mortality, dialysis dependency and reduced renal recovery [2, 3]. However, most studies which demonstrate a relationship between fluid accumulation and mortality are confounded by the fact that patients with fluid overload tend to be sicker and often need more organ support.

In general, strategies for avoiding and managing fluid overload include conservative fluid management, diuretic use and mechanical fluid removal [9–15]. A sub-analysis of the Randomized Evaluation of Normal versus Augmented Level of Renal Replacement Therapy (RENAL) study showed that achieving a negative fluid balance during continuous renal replacement therapy (CRRT) was associated with a survival benefit [11]. However, the optimal timing and best method of removing fluid and reversing fluid overload during RRT are unknown. As a result, clinical practice is variable [16]. Slower removal rates may result in prolonged exposure to the effects of fluid accumulation, including tissue oedema and progressive organ dysfunction [17, 18]. Murugan et al. analysed critically ill patients with AKI and 5% fluid overload receiving RRT and showed that an ultrafiltration intensity > 25 ml/kg/day was associated with lower 1-year mortality compared to 20 ml/kg/day [19]. More intensive ultrafiltration, however, may not be tolerated and may be associated with an increased risk of haemodynamic instability and organ dysfunction. For instance, the RENAL study showed that net ultrafiltration rates greater than 1.75 ml/kg/h compared with rates less than 1.01 ml/kg/h were associated with a reduced chance of survival [20]. Hypophosphatemia and cardiac arrhythmias were more common in patients exposed to higher ultrafiltration rates.

To reconcile the conflicting messages about ultrafiltration rates, it is important to consider whether the net effect on cumulative fluid balance may be more important than the actual ultrafiltration rate *per sé*. In addition, there is a need to identify factors which impact the chances of successful fluid removal.

The objectives of our study were to investigate the association between changes in cumulative fluid balance (FB) and mortality during CRRT and to identify relevant clinical parameters which impacted the likelihood of successful decrease of FB.

## Materials and methods

### Setting

Guy’s & St Thomas’ NHS Foundation Hospital is a tertiary care centre with a 54-bed, level 3 multidisciplinary adult intensive care unit (ICU). The ICU has a fully computerised electronic patient record system where all data are recorded at the time of generation. In our unit, CRRT is the first-line modality for all patients requiring RRT. The indications and prescription for CRRT, including FB target, are determined by the ICU team, and the treatment is routinely delivered and monitored by the ICU nursing and medical team.

### Patient and study design

We analysed prospectively collected data of adult patients admitted to the ICU between September 2012 and August 2015 who received CRRT for AKI for at least 24 h. In patients with more than 1 admission to ICU during hospitalisation, only data from the first ICU admission was analysed. Exclusion criteria were (a) age < 18 years, (b) patients with end-stage renal disease and (c) treatment with CRRT for < 24 h.

### Data collection

The following data were collected: patient demographics, age, gender, body weight (BW), height, body mass index (BMI), comorbidities and reason for admission to ICU. On the day of CRRT initiation, we collected Sequential Organ Failure Assessment (SOFA) score, haemoglobin (Hb), serum albumin, maximum arterial lactate concentration and noradrenaline data. We defined day 1 as the 24-h period, or part thereof, on which CRRT was initiated. Daily cumulative FB is calculated automatically in our electronic medical records based on total fluid intake from all sources (intravenous fluids, blood products, enteral and parenteral nutrition, and medications) minus all outputs (urine, effluent, drain losses and gastrointestinal output). We recorded daily cumulative FB from the day of ICU admission in millilitres and also as percentage body weight (% BW). The nadir of cumulative FB during CRRT was identified as the lowest cumulative FB recorded during CRRT. As an indicator of net fluid removal during CRRT, we calculated delta cumulative FB as the difference in cumulative FB between initiation of CRRT and FB nadir. To describe factors related to fluid removal practice, we recorded whether a FB target had been prescribed by the medical team and whether it was achieved in the following 24 h. Administration of fluid boluses during CRRT was used as surrogate marker of haemodynamic instability. Daily maximum arterial lactate concentration and daily SOFA score were collected as indicators of severity of illness. We also recorded whether patients had received diuretics whilst on CRRT. In patients who had had noradrenaline treatment during CRRT, we calculated the total daily dose. The outcomes of interest were ICU and hospital mortality, number of days from initiation of CRRT to lowest cumulative FB (i.e. nadir), number of days on CRRT and length of stay in ICU.

### Statistics

Categorical data were summarised as frequency (percent) and continuous data as mean and standard deviation (SD) or median and interquartile range [IQR] and compared between ICU and hospital survivors and non-survivors and in those who did and did not reach a nadir using the chi-square, Fisher’s exact, Mann-Whitney or *t* tests, as appropriate. Multivariable logistic regression was used to explore whether administration of fluid boluses, blood transfusions, diuretics, noradrenaline and setting a fluid balance target were associated with reaching a nadir. The role of cumulative fluid balance and SOFA score at RRT initiation and length of time on RRT were also considered with a backwards stepwise selection procedure used to identify variables significantly associated with reaching a nadir. Variables with *p* < 0.01 were retained in the final model.

Multivariable logistic regression was also used to explore the relationship between FB and odds of ICU and hospital survival. The model included cumulative FB at CRRT initiation and delta cumulative fluid balance (categorised as ‘no nadir’, ‘< 2.5 l’, ‘2.5 to <5 l’ or ‘5 l or more’ fluid removed). Interactions between cumulative FB and delta FB were explored, and interactions with *p* < 0.1 were added to the model. In addition, patient and disease characteristics thought *a priori* to influence survival were included in the model [age, sex, body mass index (BMI) and lactate and haemoglobin on day of CRRT, and use of noradrenaline during RRT]. This analysis was then repeated, excluding patients who did not reach a nadir, with time to FB nadir additionally included in the model and delta fluid balance included as a continuous variable. We considered the use of fractional polynomials to allow for potential non-linear relationships between mortality and both cumulative fluid balance and delta fluid balance. As there was no evidence that non-linear models provided better fit, the relationships were assumed to be linear. In sensitivity analyses, models were (1) fitted separately in patients with SOFA scores ≤ 10 and > 10 on day of initiation of CRRT, and in a subgroup of patients who (2) survived at least 4 days post CRRT initiation, and (3) had a positive FB on day of CRRT initiation.

Linear mixed effects models were used to explore differences in the rate of change of cumulative fluid balance in hospital survivors and non-survivors. Analysis was conducted using Stata 15/IC.

## Results

During the study period, 820 patients met the inclusion criteria and were analysed (median age 65 years; 49% female) (Table 1). At CRRT initiation, the median cumulative FB was + 1772 ml; 89 patients (10.9%) had a cumulative FB > 10% BW.

**Table 1:** Patient characteristics and clinical parameters

Parameter	All patients (*n* = 820)
**Demographics**	
Age, median [IQR]	65 [52–75]
Male gender, *n* (%)	511 (62)
BMI, *n* (%)	
< 20	34 (4)
20 to < 25	225 (29)
25 to < 30	255 (33)
30 to < 40	200 (26)
40 or more	53 (7)
**Reason for ICU admission**	
Medical (%)	73.2
Surgical (%)	26.8
**Admission diagnosis**	
Sepsis (%)	38.7
Post cardiac surgery (%)	4.1
Post vascular surgery (%)	6.7
Post non-cardiovascular surgery (%)	9.5
Cardiac arrest (%)	3.1
Cardiac emergency without cardiac arrest (%)	14.5
Neurological emergency (%)	2.3
Gastrointestinal emergency (%)	6.7
Acute kidney injury (%)	3.6
Decompensated liver disease (%)	4.6
Others (%)	6.2
**Comorbidities (%)**	
Hypertension	56.4
Heart disease	49.5
Diabetes mellitus	35.6
Chronic kidney disease	43.3
Cancer	20.2
Chronic obstructive pulmonary disease	18.4
Chronic liver disease	14.2
**Parameters at initiation of CRRT**	
SOFA score, mean (SD)	10.4 (3.8)
Arterial lactate concentration [mmol/l], median [IQR]	3 [1.8–6.5]
Treatment with vasopressor, *n* (%)	588 (72)
Hb [g/dl], median [IQR]	9.8 [8.8–11]
Cumulative fluid balance [ml], median [IQR]	1772 [336–4536]
**Outcomes**	
ICU mortality, *n* (%)	264 (32)
Hospital mortality, *n* (%)	331 (40)

*Abbreviations*: *BMI* body mass index, *ICU* intensive care unit, *IQR* interquartile range, *CRRT* continuous renal replacement therapy, *SOFA* Sequential Organ Failure Assessment, *Hb* haemoglobin, *SD* standard deviation

ICU mortality was 32%, and hospital mortality was 40%. Hospital survivors had a significantly lower cumulative FB at initiation of CRRT compared to patients who died (1495 versus 2184 ml; *p* < 0.001) (Table 2). Among survivors, there was also a significantly lower proportion of patients with a cumulative FB > 5% BW, > 10% BW or FB greater than the median value. Patients with a cumulative FB above the median value (1772 ml) at CRRT initiation were older, had a significantly higher SOFA score and higher arterial lactate concentration and were more likely to be on vasopressors at CRRT initiation compared to those with a lower cumulative FB (Supplementary Table S1).

**Table 2:** Cumulative fluid balance in survivors and non-survivors

Parameters	ICU non-survivors (*n* = 264**)	ICU survivors (*n* = 556**)	***p*** value	Hospital non-survivors (*n* = 331**)	Hospital survivors (*n* = 489**)	***p*** value
**Cumulative FB on day of CRRT initiation**						
**Cum FB** (ml) *	2170 [739–5451]	1581 [263–4254]	< 0.001	2184 [707–5323]	1495 [238–404]	< 0.001
**Cum FB** (% BW)*	2.9 [0.9–7.2]	2.1 [0.3–5.7]	0.001	2.9 [0.9–7.2]	2.0 [0.3–5.4]	< 0.001
**Cum FB ≤ 5% BW**, *n* (%)	167 (30)	390 (70)	0.034	211 (38)	346 (62)	0.032
**Cum FB > 5% BW**, *n* (%)	90 (38)	149 (62)		110 (46)	129 (54)	
**Cum FB ≤ 10% BW**, *n* (%)	222 (31)	485 (69)	0.132	276 (39)	431 (61)	0.037
**Cum FB > 10% BW**, *n* (%)	35 (39)	54 (61)		45 (51)	44 (49)	
**Cum FB ≤ median FB** (1772 ml), *n* (%)	112 (27)	298 (73)	0.003	142 (35)	268 (65)	0.001
**Cum FB > median FB** (1772 ml), *n* (%)	152 (37)	258 (63)		189 (46)	221 (54)	
**Cumulative FB at nadir during CRRT**						
**Cum FB** (ml) *	1115 [− 656 to 3493]	− 275 [− 4401 to 1714]	< 0.001	990 [− 1078 to 3239]	−361 [− 4729 to –1709]	< 0.001
**Cum FB** (% BW)*	1.6 [− 0.8 to 4.7]	− 0.3 [− 4.9 to –2.5]	< 0.001	1.2 [− 1.6 to 4.0]	−0.6 [− 5.5 to –2.1]	< 0.001
**Delta cumulative FB (i.e. maximum change in cumulative FB between initiation of CRRT and nadir)**						
**Delta FB** (ml) *	541 [0–3461]	2479 [183–6242]	< 0.001	882 [0–3651]	2688 [286–6512]	< 0.001
**Delta FB** (% BW)*	0.9 [0–4.7]	3.1 [0.3–7.7]	< 0.001	1.4 [0–5.0]	3.3 [0.3–8.0]	< 0.001
Time to nadir of cumulative FB***						
**1 to 3 days**	54 (26)	152 (74)	0.746	73 (35)	133 (65)	0.577
**> 3 days**	98 (25)	294 (75)		130 (33)	262 (67)	

*Abbreviations*: *BW* body weight, *FB* fluid balance, *Cum FB* cumulative fluid balance, *ICU* intensive care unit, *IQR* interquartile range, *CRRT* continuous renal replacement therapy

*Median [interquartile range]

**Weight was unknown for 7 ICU non-survivors, 17 ICU survivors, 10 hospital non-survivors and 14 hospital survivors; therefore, the fluid balance as per cent of body weight was not calculated for these patients

***Survival compared by time to nadir in *n* = 598 patients who reached a nadir

### Prescription of fluid balance target

Among all 820 patients, a FB target was set on every day of CRRT for 38% of patients and on at least 80% of CRRT days for 70% of patients (Table 3). Two per cent of patients did not have a daily FB target prescribed on any day of CRRT. Their ICU mortality was higher compared to patients with a FB target prescribed every day. On the days when a FB target was set, the median net FB on the following morning was − 105 ml (IQR − 871 to 538) compared to + 585 ml (IQR − 225 to1745) on days without a prescribed FB target.

**Table 3:** Prescription of daily fluid balance target during CRRT and outcomes

	Proportion of days when a daily FB target was set				
	0%	> 0 to < 50%	50 to < 80%	80 to < 100%	100%
***n*** (%)	13 (2%)	27 (3%)	201 (25%)	266 (32%)	313 (38%)
**Age**, median [IQR]	58 [51–74]	65 [55–74]	66 [52–74]	63 [50–73]	67 [53–76]
**SOFA score at CRRT initiation**, mean (SD)	14.3 (5.1)	11.9 (4.7)	10.1 (4.0)	10.8 (3.5)	9.8 (3.6)
**Maximum SOFA score during CRRT**, mean (SD)	16.3 (4.5)	14.7 (4.4)	12.4 (4.0)	13.9 (3.0)	11.7 (3.7)
**No FB nadir during CRRT**, *n* (%)	12 (92%)	23 (81%)	74 (37%)	44 (17%)	70 (22%)
**One or more fluid boluses received during CRRT**, *n* (%)	11 (85%)	21 (78%)	151 (75%)	238 (89%)	202 (65%)
**Blood transfusion received during CRRT**, *n* (%)	10 (77%)	21 (78%)	134 (67%)	243 (91%)	214 (68%)
**Any diuretics received during CRRT**, *n* (%)	4 (31%)	9 (33%)	93 (46%)	152 (57%)	178 (57%)
**Any noradrenaline received during RRT**, *n* (%)	8 (62%)	19 (70%)	141 (70%)	231 (87%)	210 (37%)
**Percentage of days when noradrenaline was given during CRRT**, median [IQR]	100 [0–100]	100 [0–100]	50 [0–100]	42 [20–80]	42 [0–80]
**Daily noradrenaline dose** [μg], mean*					
**0**	5 (39%)	8 (30%)	60 (30%)	35 (13%)	103 (33%)
**1–4999**	0	5 (19%)	18 (9%)	42 (16%)	37 (12%)
**5000–9999**	0	0	26 (13%)	33 (12%)	31 (10%)
**10,000–49,999**	2 (15%)	5 (19%)	71 (35%)	139 (52%)	105 (34%)
***≥*** **50,000**	6 (46%)	9 (33%)	26 (13%)	17 (6%)	37 (12%)
**Days on CRRT**, median [IQR]	2 [2–3]	3 [3–6]	4 [3–8]	14 [8–24]	5 [4–9]
**ICU mortality**, *n* (%)	12 (92%)	20 (74%)	74 (32%)	85 (32%)	73 (23%)

*Abbreviations*: *CRRT* continuous renal replacement therapy, *FB* fluid balance, *ICU* intensive care unit, *IQR* interquartile range, *SOFA* Sequential Organ Failure Assessment, *SD* standard deviation

*Mean daily dose across all days on RRT in mg

On 51% of CRRT days, the achieved net FB was in the range of the prescribed FB target (Supplementary Table S2). On days when patients did not achieve a FB as prescribed, they had also received more fluid boluses and/or blood transfusions and a higher daily dose of noradrenaline, and their SOFA score and maximum arterial lactate concentration were higher (Table 4).

**Table 4:** Disease severity and interventions on CRRT days when FB targets were and were not achieved

Interventions/clinical status in the 24-h period	Net FB target met	Prescribed fluid balance target						
		> 1 l positive	Up to 1 l positive	FB neutral	Up to 1 l negative	1 to 2 l negative	> 2 l negative	All
One or more fluid boluses received	No	3/14 (21%)	306/544 (56%)	553/1508 (37%)	305/823 (37%)	252/664 (38%)	61/167 (37%)	1480/3720 (40%)
	Yes	75/99 (76%)**	220/615 (36%)**	173/980 (18%)**	233/1289 (18%)**	108/661 (16%)**	50/281 (18%)**	859/3925 (22%)**
Blood transfusion received	No	6/14 (43%)	249/544 (46%)	590/1508 (39%)	294/823 (36%)	205/664 (31%)	35/167 (21%)	1379/3720 (37%)
	Yes	53/99 (54%)**	197/615 (32%)**	234/980 (24%)**	335/1289 (26%)**	101/661 (15%)**	29/281 (10%)**	949/3925 (24%) **
Fluid bolus and/or blood transfusion	No	8/14 (57%)	388/544 (71%)	870/1508 (58%)	457/823 (56%)	369/664 (56%)	79/167 (47%)	2171/3720 (58%)
	Yes	84/99 (85%)**	325/615 (53%)**	346/980 (35%)**	483/1289 (37%)**	186/661 (28%)**	74/281 (26%)**	1498/3925 (38%)**
Any diuretic received	No	4/14 (29%)	97/544 (18%)	253/1508 (17%)	153/823 (19%)	108/664 (16%)	17/167 (10%)	632/3720 (17%)
	Yes	19/99 (19%)	90/615 (15%)	120/980 (12%)	177/1289 (14%)**	81/661 (12%)*	32/281 (11%)	519/3925 (13%)
Any noradrenaline received	No	7/14 (50%)	345/544 (63%)	727/1508 (48%)	355/823 (43%)	298/664 (45%)	70/167 (42%)	1802/3720 (48%)
	Yes	73/99 (74%)	330/615 (54%)*	422/980 (43%)*	537/1289 (42%)	239/661 (36%)**	108/281 (38%)	1709/3925 (44%)
Total noradrenaline dose received [μg]***, median [IQR]	No	7936 [4160–44,010]	20,992 [9648–46,480]	20,032 [7680–43,023]	15,242 [5760–32,224]	9264 [3264–22,203]	7743 [1760–26,432]	16,800 [6000–36,720]
	Yes	49,280 [14688–94,624]	16,800 [6280–37,248]*	14,144 [4312–32,408]*	11,360 [3840–26,447]*	8360 [2640–18,695]	6200 [2360–17,524]	12,760 [4080–29,776]**
Maximum lactate [mmol/l], median [IQR]	No	2.3 [1.5–3.9]	2.6 [1.8–4.2]	2.2 [1.6–3.3]	2.1 [1.6–2.9]	2.0 [1.6–2.8]	1.9 [1.5–2.5]	2.2 [1.6–3.1]
	Yes	5.6 [3.7–8.9]**	2.3 [1.8–3.3]**	1.9 [1.5–2.5]**	2.0 [1.6–2.6]**	1.9 [1.5–2.4]**	1.8 [1.5–2.4]	2 [1.5–2.7]**
SOFA score, mean (SD)	No	10.3 (3.3)	10.9 (3.4)	9.9 (3.6)	10.1 (3.4)	10.0 (3.6)	9.8 (3.4)	10.1 (3.5)
	Yes	12.4 (2.9)*	9.9 (3.3)**	9.4 (3.3)**	9.6 (3.3)**	9.1 (3.3)**	9.6 (3.7)	9.6 (3.4)**

*Abbreviations*: *FB* fluid balance, *IQR* interquartile range, *SD* standard deviation, *SOFA* Sequential Organ Failure Assessment

FB after 24 h was considered to be in target if it fell within the following ranges: target > 1 l positive: net FB range > 0.9 l; target up to 1 l: net FB range 0.5 to 1.1 l; target FB neutral: net FB range − 0.25 to 0.25 l; target FB up to 1 l negative: net FB − 0.05 to − 1.1 l; target 1 l to 2 l negative: net FB − 1 to − 2.1 l; target > 2 l negative: net FB > − 2 l

*p < 0.05, **p < 0.001, ***on days when noradrenaline was received

### Fluid balance during CRRT

**Fig. 1:**
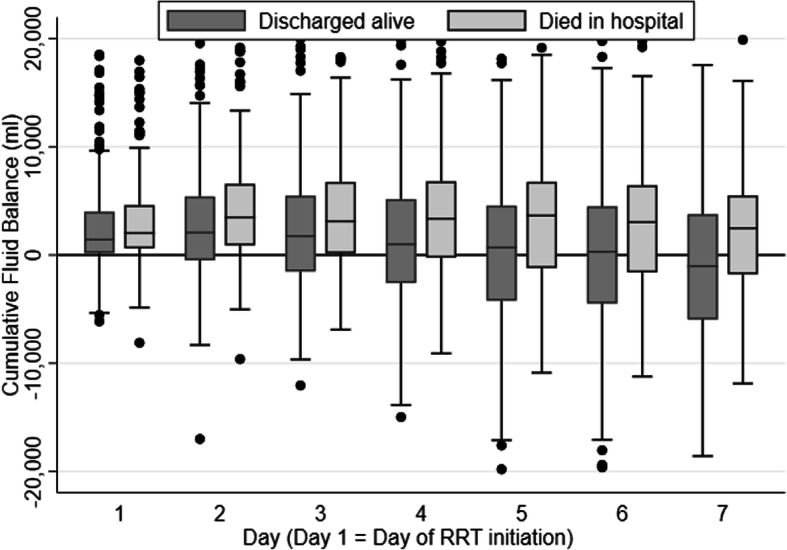
Trends in cumulative fluid balance during RRT. RRT renal replacement therapy

The nadir of cumulative FB during CRRT was significantly lower in hospital survivors than in non-survivors (− 361 ml versus + 990 ml; *p* < 0.001) (Table 2). As such, net fluid removal and delta FB (i.e. difference in cumulative fluid balance between CRRT initiation and day of nadir) were significantly larger in survivors. Univariate analysis showed that patients who were on vasopressor therapy at CRRT initiation had a significantly lower net decrease of cumulative FB compared to patients not receiving vasopressor support [median net decrease 1544 ml (IQR 0–4957) versus 2598 ml (IQR 504–6080); *p* = 0.001]. There was a significant difference in the rate of change of cumulative FB among hospital survivors and non-survivors (*p* = 0.003) (Fig. 1). In the 7 days after CRRT initiation, the cumulative FB declined by an average of 463 ml per day [95% confidence interval (CI) 314 to 612, *p* < 0.001) in survivors, whilst there was no significant change in non-survivors [mean decline = 110 ml per day (95% CI − 57 to 277), *p* = 0.195].

In 221 patients, the cumulative FB did not decrease during CRRT and they did not reach a FB nadir (Supplementary Table S3). Higher severity of illness at initiation of CRRT, shorter duration of CRRT, more days on CRRT without a prescribed FB target and need for higher doses of noradrenaline were independent risk factors for not reaching a FB nadir during CRRT (Table 5).

**Table 5:** Risk factors for not reaching a fluid balance nadir during CRRT

	Univariable analysis			Multivariable analysis	
	FB nadir reached during CRRT (***n*** = 598)	FB nadir not reached during CRRT (***n*** = 222)	***p*** value	OR 95% CI	***p*** value
**Cumulative FB at CRRT initiation**	1.7 [0.3–4.6]	2.0 [0.7–4.5]	0.055		0.491
**SOFA score at CRRT initiation**	9.8 (3.6)	11.9 (4.1)	< 0.001	1.17 (1.12–1.23)	< 0.001
**Days on CRRT**	8 [5–16]	5 [3–7]	< 0.001	0.93 (0.90–0.95)	< 0.001
**Proportion of days on which a FB target was set**	0.92 [0.80–1.00]	0.80 [0.60–1.00]	< 0.001	0.77 (0.70–0.84)	< 0.001
**Administration of one or more fluid boluses during RRT**	428 (72%)	195 (88%)	< 0.001		0.754
**Administration of blood transfusion during RRT**	451 (75%)	171 (77%)	0.632		0.123
**Administration of one or more doses of diuretics during RRT**	331 (55%)	105 (47%)	0.040		0.750
**Daily noradrenaline dose** [μg], mean*					
**0**	145 (24%)	66 (30%)	< 0.001	1	< 0.001
**1–4999**	166 (28%)	27 (12%)		0.47 (0.27–0.81)	
**5000–9999**	76 (13%)	11 (5%)		0.46 (0.22–0.97)	
**10,000–49,999**	189 (32%)	78 (35%)		1.07 (0.68–1.68)	
***≥*** **50,000**	22 (4%)	40 (18%)		2.59 (1.31–5.14)	

*Abbreviations*: *CI* confidence interval, *CRRT* continuous renal replacement therapy, *FB* fluid balance, *ICU* intensive care unit, *OR* odds ratio, *SOFA* sequential organ failure assessment

Values expressed as *n* (%), mean (standard deviation) or median [interquartile range]. C-statistic 0.76, Hosmer Lemeshow goodness of fit: *p* = 0.21

*Mean daily dose across all days on RRT

In patients with a fall in cumulative FB during CRRT, the median time to FB nadir was 5 days (Supplementary Table S3). In a multivariable analysis, younger age, lower SOFA score, higher haemoglobin, lower arterial lactate concentration and not requiring vasopressor support at CRRT initiation were independently associated with a shorter time to reaching the cumulative FB nadir. There was no significant difference in duration from 1st day of RRT to FB nadir between patients with a cumulative FB above and below the median value or patients with a cumulative FB below or above 10% BW (Supplementary Table S3).

### Multivariable analyses

Multivariable analysis showed that older age, higher SOFA score, need for higher daily doses of noradrenaline and lower net fluid loss during CRRT were independently associated with ICU mortality (Table 6). Independent risk factors for hospital mortality were older age, higher SOFA score at initiation of CRRT, lower BMI, need for higher doses of noradrenaline and lower net fluid loss on CRRT initiation. Cumulative FB was not independently associated with mortality (Table 6). There was no evidence of an interaction between cumulative FB at the start of CRRT and fluid loss (*p* = 0.66), and the association between volume of fluid removal and outcome was also independent of cumulative FB at CRRT initiation (OR 1.01, CI 0.97–1.05; *p* = 0.59).

**Table 6:** Multivariable analysis

	ICU mortality			Hospital mortality		
	OR	95% CI	*p* value	OR	95% CI	*p* value
Age	1.02	1.01–1.03	< 0.001	1.02	1.01–1.03	0.001
Male sex	1.21	0.84–1.73	0.30	1.23	0.89–1.70	0.23
BMI						
< 20	2.43	1.01–5.82		3.09	1.32–7.23	
20 to < 25	1		0.22	1		0.037
25 to < 30	0.90	0.59–1.39		0.95	0.64–1.43	
30 to < 40	0.84	0.53–1.31		0.86	0.57–1.31	
40 or higher	0.86	0.42–1.78		1.53	0.79–2.97	
SOFA score on the 1st day of CRRT	1.17	1.11–1.23	< 0.001	1.13	1.08–1.16	< 0.001
Highest arterial lactate concentration on 1st day of CRRT (μmol/l)	1.03	0.98–1.07	0.23	1.04	1.00–1.08	0.065
Hb on 1st day of CRRT (per 10 g/dl)	0.99	0.90–1.09	0.86	0.98	0.89–1.07	0.61
Daily noradrenaline dose [μg], mean*						
0	1		< 0.001	1		0.002
1–4999	0.87	0.50–1.51		0.92	0.56–1.51	
5000–9999	0.96	0.48–1.93		1.12	0.62–2.03	
10,000–49,999	2.60	1.62–4.17		1.94	1.25–3.00	
≥ 50,000	2.77	1.40–5.50		2.10	1.06–4.19	
Cumulative FB at CRRT initiation (per 1000 ml)	1.01	0.97–1.05	0.80	1.01	0.97–1.05	0.59
Delta cumulative FB						
No nadir reached	1		0.048	1		0.010
< 2500 ml reduction	0.84	0.48–1.46		0.77	0.54–1.29	
2500–4999 ml reduction	0.57	0.36–0.91		0.67	0.43–1.03	
≥ 5000 ml reduction	0.57	0.33–0.99		0.43	0.26–0.71	

*Abbreviations*: *BW* body weight, *BMI* body mass index, *CI* confidence interval, *CRRT* continuous renal replacement therapy, *FB* fluid balance, *Hb* haemoglobin in [g/dl], *ICU* intensive care unit, *IQR* interquartile range, *OR* odds ratio, *SOFA* Sequential Organ Failure Assessment

*Mean daily dose across all days on RRT

There were no significant interactions between cumulative and delta fluid balance (ICU model: *p* = 0.87, hospital model: *p* = 0.66). C-statistic: ICU model c = 0.77, hospital model c = 0.73, Hosmer Lemeshow goodness of fit: ICU model: *p* = 0.31, hospital model: *p* = 0.33

We repeated the multivariable analysis and excluded patients who did not reach a nadir FB (Supplementary Table S4). Independent risk factors for hospital mortality were older age, lower BMI, higher SOFA score, need for higher doses of noradrenaline and lower net fluid removal between CRRT initiation and FB nadir. Cumulative FB at initiation of CRRT and time to nadir were not independent risk factors. We also repeated the multivariable analysis after exclusion of patients with a negative cumulative FB at CRRT initiation (Supplementary Table S5). Independent risk factors for hospital mortality were older age, lower BMI, and higher SOFA score and arterial lactate concentration at initiation of CRRT and need for higher doses of noradrenaline.

### Sensitivity analyses

We differentiated between patients with SOFA score ≤ 10 and > 10 at CRRT initiation (Supplementary Table S6). Cumulative FB at initiation of CRRT was not independently associated with risk of mortality in ICU or hospital in either group. Among patients with a higher SOFA score on the day of CRRT initiation, those without a decline in cumulative FB had a significantly higher mortality.

To correct for the fact that in patients who died early, there were only a few days in which fluid could be removed and the cumulative FB nadir could be reached, we repeated the analysis and excluded patients who died before day 5 of CRRT. The analysis showed very similar results to the main analysis shown in Table 6.

## Discussion

Fluid management in AKI is challenging [15, 16]. The main findings of our study were as follows: First, in critically ill adult patients, cumulative FB at the time of initiation of acute CRRT was not independently associated with mortality after correction for severity of illness and need for vasopressor support. Instead, a decline of cumulative FB was independently associated with survival. Second, patients who reached a lower fluid balance nadir during CRRT were more likely to survive. Third, a higher proportion of days without a prescribed FB target and administration of fluid boluses during CRRT were independently associated with failure to reach a FB nadir. The time to FB nadir was not independently associated with an increased risk of mortality.

Our results are in line with some results of previous studies but not all. Investigators of the Program to Improve Care in Acute Renal Disease (PICARD) study showed that greater fluid overload on the first day of RRT was associated with a higher risk of dying [2]. However, they did not correct for severity of illness on the day of RRT initiation. Woodward et al. recently analysed the data of 481 patients receiving CRRT [3]. Two hundred thirty-eight patients (49.5%) had a cumulative FB > 10% BW at CRRT initiation. This group had a significantly higher risk of an adverse 90-day composite major kidney event, a composite outcome consisting of all-cause mortality, CRRT dependence, or an inability to recover more than 50% of baseline estimated glomerular filtration up to 90 days following hospital discharge. The authors reported quick SOFA results and vasopressor use on the day of CRRT initiation but did not correct for any other parameters of acute severity of illness at CRRT initiation which may explain the difference between these results and our finding. In addition, the proportion of patients with cumulative FB > 10% BW was much higher than in our cohort (49.5% versus 10.9%).

Our finding that patients who tolerated fluid removal during CRRT and reached a cumulative FB nadir had a better outcome than patients who did not reach a FB nadir is not surprising. Not being able to tolerate fluid removal is generally a sign of severity of illness. Our results also emphasise the importance of setting a FB target as part of the daily CRRT prescription. Although the prescribed FB target was only achieved on 52% of CRRT days, the proportion of days with a prescribed FB target was independently associated with a higher chance of achieving a FB nadir during CRRT.

A subgroup analysis of the RENAL study concluded that a negative fluid balance during CRRT was associated with an improved survival [11]. Murugan et al. demonstrated that a higher ultrafiltration intensity was also associated with a lower mortality at 1 year [17]. In contrast, in paediatric patients treated with CRRT, Goldstein et al. found no difference in net fluid removal between survivors and non-survivors [21]. To reconcile these findings, the impact on cumulative FB may have more impact on outcome than ultrafiltration rate in isolation.

Our data have important implications for clinical practice. Although we did not find an independent association between cumulative FB at initiation of CRRT and outcome, we showed that net fluid removal was independently associated with lower mortality. We would argue that every attempt should be made to decrease cumulative FB during CRRT. Prescribing a net FB target is one of the key factors which determines the likelihood of achieving a FB nadir. However, we acknowledge that evidence-based data guiding the practice of fluid removal are lacking and that clinical practice is variable [16]. In our opinion, intervention studies in this area are urgently needed.

Despite the important findings of our study, we would like to acknowledge some limitations. This is a retrospective single-centre study of a heterogeneous patient population receiving CRRT as prescribed and delivered by the clinical team. Nevertheless, with patients typical of an ICU population in a tertiary care centre, we feel that our data are representative of a large proportion of patients admitted to ICUs worldwide. Second, we only evaluated the impact of fluid balance in AKI patients treated with CRRT but did not analyse AKI patients who received diuretic treatment for fluid overload. Third, like many observational studies on this subject, we did not account for pre-admission FB or insensible fluid losses when determining cumulative FB. Furthermore, we evaluated cumulative FB as the difference between all inputs and outputs but did not explicitly determine intravascular volume status. Fourth, in our unit, mechanical fluid removal is managed by the critical care staff without a standardised target-driven protocol. As such, there might be variation in the clinical management of fluid removal. Fifth, we used fluid boluses during CRRT as a surrogate marker for haemodynamic instability but did not explore changes in vasopressor dose. Sixth, we investigated the association between fluid balance and hospital mortality but did not analyse any other outcomes, including outcome at 1 year or renal recovery. Finally, the study was non-interventional and the association between cumulative fluid balance and outcome does not prove a causal relationship.

## Conclusion

Our study highlights the importance of fluid management during CRRT. A decline of cumulative FB during CRRT was associated with a lower risk of mortality. Intervention studies are necessary to confirm these findings.

## Supplementary information

**Additional file 1 : Table S1**. Comparison between patients with cumulative fluid balance below and above the median at initiation of CRRT.

**Additional file 2 : Table S2.** Comparison between prescribed fluid balance target and achieved net fluid balance.

**Additional file 3 : Table S3.** Time between initiation of CRRT and nadir of cumulative fluid balance depending on initial cumulative fluid balance.

**Additional file 4 : Table S4.** Multivariable analysis (excluding patients who did not meet a FB nadir).

**Additional file 5 : Table S5.** Multivariable analysis (excluding 116 patients with negative cumulative fluid balance of day of initiation of CRRT).

**Additional file 6 : Table S6.** Multivariable analysis based on SOFA score at CRRT initiation.

## Data Availability

The datasets used and analysed during the current study are available from the corresponding author on reasonable request.

## Abbreviations

AKI: Acute kidney injury
BMI: Body mass index
BW: Body weight
CI: Confidence interval
CKD: Chronic kidney disease
CRRT: Continuous renal replacement therapy
Cum FB: Cumulative fluid balance
ESRD: End-stage renal disease
FB: Fluid balance
Hb: Haemoglobin
ICU: Intensive care unit
IQR: Interquartile ratio
OR: Odds ratio
PICARD: Program to Improve Care in Acute Renal Disease
REC: Research Ethics Committee
RRT: Renal replacement therapy
SD: Standard deviation
SOFA: Sequential Organ Failure Assessment
