# Supplementary material for: Fluid removal associates with better outcomes in critically ill patients receiving continuous renal replacement therapy: a cohort study
Source: Crit Care. 2020 Jun 1;24:279. doi: 10.1186/s13054-020-02986-4 (PMC7268712; doi:10.1186/s13054-020-02986-4)
Supplement: Supplementary file 1 — Additional file 1 : Table S1. Comparison between patients with cumulative fluid balance below and above the median at initiation of CRRT. [file 13054_2020_2986_MOESM1_ESM.docx]

**Supplementary Table S1 Comparison between patients with cumulative fluid balance below and above the median at initiation of CRRT**

| **Parameters at initiation of CRRT** | **All patients**  (n=820) | **Patients with cum FB ≤ median value**  (n=410) | **Patients with cum FB > median value**  (n=410) | **p-value** |
| --- | --- | --- | --- | --- |
| **Age**, median [IQR] | 65 [52,75] | 62.5 [50,73] | 67 [53,77] | 0.009 |
| **Male gender**, n (%) | 511 (62) | 262 (64) | 649 (61) | 0.273 |
| **SOFA score**, mean (SD) | 10.4 (3.8) | 9.6 (4.1) | 11.1 (3.4) | <0.001 |
| **Arterial lactate concentration**, median [IQR] | 3 [1.8;6.5] | 2.7 [1.7,6.1] | 3.4 [2.1,6.6] | 0.001 |
| **On vasopressor support**, n (%) | 588 (72) | 278 (68) | 310 (76) | 0.013 |
| **Hemoglobin** [g/dL], median [IQR] | 9.8 [8.8,11] | 10.0 [8.8,11.2] | 9.8 [8.9,11.0] | 0.843 |

Abbreviations: Cum FB = cumulative FB; CRRT = continuous renal replacement therapy; SOFA = Sequential Organ Failure Assessment; SD = standard deviation; IQR = interquartile range
