# Supplementary material for: Fluid removal associates with better outcomes in critically ill patients receiving continuous renal replacement therapy: a cohort study
Source: Crit Care. 2020 Jun 1;24:279. doi: 10.1186/s13054-020-02986-4 (PMC7268712; doi:10.1186/s13054-020-02986-4)
Supplement: Supplementary file 2 — Additional file 2 : Table S2. Comparison between prescribed fluid balance target and achieved net fluid balance. [file 13054_2020_2986_MOESM2_ESM.docx]

**Supplementary Table S2: Comparison between prescribed fluid balance target and achieved net fluid balance**

| **Prescribed FB target for next 24 hours** | **Number of days** | **Achieved net fluid balance 24 hours later (in litres) *** | **Achieved net FB in range of prescribed FB target **** |
| --- | --- | --- | --- |
| >1L positive | 113 (1%) | +2.6 [1.5 to 3.8] | 99 (88%) |
| up to 1L positive | 1159 (15%) | +0.6 [0.3 to 1.3] | 615 (53%) |
| FB neutral | 2488 (33%) | +0.1 [-0.1 to 0.8] | 980 (39%) |
| up to 1L negative | 2112 (28%) | -0.5 [-0.8 to 0] | 1289 (61%) |
| 1L to 2L negative | 1325 (17%) | -1.1 [-1.5 to -0.7] | 661 (50%) |
| > 2L negative | 448 (6%) | -2.2 [-3.0 to -1.6] | 281 (63%) |
| ALL | 7645 | -0.1 [-0.9 to 0.5] | 3925 (51%) |

Abbreviations: FB = fluid balance

* median and interquartile range

** Fluid balance after 24hrs was considered to be in target if it fell within the following ranges: target >1L positive: net FB range >0.9L

target up to 1L: net FB range 0.5 to 1.1L;

target FB neutral: net FB range -0.25 to 0.25L;

target FB up to 1L negative: net FB -0.05 to -1.1L

target 1L to 2L negative: net FB -1 to -2.1L

target > 2L negative: net FB >-2L
