# Supplementary material for: Fluid removal associates with better outcomes in critically ill patients receiving continuous renal replacement therapy: a cohort study
Source: Crit Care. 2020 Jun 1;24:279. doi: 10.1186/s13054-020-02986-4 (PMC7268712; doi:10.1186/s13054-020-02986-4)
Supplement: Supplementary file 3 — Additional file 3 : Table S3. Time between initiation of CRRT and nadir of cumulative fluid balance depending on initial cumulative fluid balance. [file 13054_2020_2986_MOESM3_ESM.docx]

**Supplementary Table S3 Time between initiation of CRRT and nadir of cumulative fluid balance depending on initial cumulative fluid balance**

| **Time to cumulative FB nadir** | **All patients** | **Patients with cum FB ≤ median value at initiation of CRRT**  **(n=410)** | **Patients with cum FB > median value at initiation of CRRT**  **(n=410)** | **p-value** |
| --- | --- | --- | --- | --- |
| **No nadir reached**, n (%) | 221 (27) | 102 (25) | 119 (29) | 0.355 |
| **1 to 3 days**, n (%) | 206 (25) | 109 (27) | 97 (24) |  |
| **>3 days**, n (%) | 393 (48) | 199 (49) | 194 (47) |  |
| **Time to cum FB nadir ***, median [IQR] | 5 [3 - 10] | 5 [2 - 10] | 5 [3 - 9] | 0.847 |
|  |  |  |  |  |
|  |  | **Patients with cum FB ≤ 10% BW at CRRT initiation ****  **(n=707)** | **Patients with cum FB > 10% BW at CRRT initiation ****  **(n=89)** | **p-value** |
| **No nadir reached**, n (%) | 216 (27) | 194 (27) | 22 (25) | 0.251 |
| **1 to 3 days**, n (%) | 196 (25) | 179 (25) | 17 (19) |  |
| **>3 days**, n (%) | 384 (48) | 334 (47) | 50 (56) |  |
| **Time to cum FB nadir ***, median (IQR) | 5 [3 - 10] | 5 [3 - 9] | 6 [3 - 10] | 0.189 |

Abbreviations: BW = body weight; cum FB = cumulative FB; CRRT = continuous renal replacement therapy; IQR = interquartile range

* excluding patients with lowest FB on day of CRRT

** 24 patients with unknown weight were excluded from analysis of cumulative fluid balance as a percent of body weight
