# Supplementary material for: Fluid removal associates with better outcomes in critically ill patients receiving continuous renal replacement therapy: a cohort study
Source: Crit Care. 2020 Jun 1;24:279. doi: 10.1186/s13054-020-02986-4 (PMC7268712; doi:10.1186/s13054-020-02986-4)
Supplement: Supplementary file 4 — Additional file 4 : Table S4. Multivariable analysis (excluding patients who did not meet a FB nadir). [file 13054_2020_2986_MOESM4_ESM.docx]

**Supplementary Table S4: Multivariable analysis (excluding patients who did not meet a FB nadir)**

|  | **ICU mortality** | | | **Hospital mortality** | | |
| --- | --- | --- | --- | --- | --- | --- |
|  | **OR** | **95% CI** | **p-value** | **OR** | **95% CI** | **p-value** |
| **Age** | 1.03 | 1.01 – 1.04 | <0.001 | 1.02 | 1.01 – 1.03 | 0.008 |
| **Male sex** | 1.36 | 0.88 – 2.10 | 0.17 | 1.34 | 0.90 – 1.99 | 0.14 |
| **BMI**  **<20**  **20 to <25**  **25 to <30**  **30 to <40**  **40** | 3.08  1  0.79  0.80  1.10 | 0.99 – 9.55  0.46 – 1.35  0.47 – 1.36  0.49 – 2.52 | 0.18 | 4.17  1  1.08  0.93  1.76 | 1.44 – 12.12  0.67 – 1.76  0.57 – 1.52  0.83 – 1.09 | 0.049 |
| **SOFA score on 1^st^ day of CRRT** | 1.12 | 1.04 – 1.20 | <0.001 | 1.06 | 1.00 – 1.12 | 0.067 |
| **Highest arterial lactate concentration on 1^st^ day of CRRT** (µmol/L) | 0.98 | 0.92 - 1.04 | 0.50 | 1.01 | 0.95 - 1.06 | 0.84 |
| **Daily noradrenaline dose** [µg], mean *****  **0**  **1 – 4,999**  **5,000 – 9,999**  **10,000 – 49,999**  $\boldsymbol{\geq}$**50,000** | 1  1.39  1.49  3.85  3.22 | 0.69 – 2.81  0.65 – 3.42  1.99 – 7.44  1.11 – 9..30 | <0.001 | 1  1.83  1.77  3.28  3.75 | 1.00 – 3.36  0.87 – 3.65  1.82 – 5.93  1.36 – 10.31 | 0.001 |
| **Hb on 1^st^ day of CRRT** (per 10g/dL) | 1.00 | 0.88 - 1.13 | 0.98 | 0.97 | 0.87 - 1.09 | 0.63 |
| **Cumulative FB at CRRT initiation** (per 1000ml) | 1.01 | 0.96 - 1.07 | 0.64 | 1.03 | 0.98 - 1.07 | 0.30 |
| **Delta cumulative FB** (per 1000ml) | 0.96 | 0.90 – 1.01 | 0.10 | 0.95 | 0.91 - 0.99 | 0.024 |
| **Time to cumulative FB nadir**  **1 to 3 days**  **>3 days** | 1  0.94 | 0.58 - 1.54 | 0.81 | 1  0.96 | 0.61 - 1.50 | 0.85 |

Abbreviations: BW = body weight; BMI = body mass index; CI = confidence interval; FB = fluid balance; Hb = haemoglobin in [g/dL]; ICU = intensive care unit; IQR = interquartile range; OR = odds ratio; CRRT = continuous renal replacement therapy; SOFA = Sequential Organ Failure Assessment. * mean daily dose across all days on RRT

There were no significant interactions between cumulative and delta fluid balance (ICU model: p=0.99, hospital model: p=0.96).

C-statistic: ICU model c=0.74, hospital model c=0.71, Hosmer Lemeshow goodness of fit: ICU model: p=0.55, hospital model: p=0.36
