# Supplementary material for: Fluid removal associates with better outcomes in critically ill patients receiving continuous renal replacement therapy: a cohort study
Source: Crit Care. 2020 Jun 1;24:279. doi: 10.1186/s13054-020-02986-4 (PMC7268712; doi:10.1186/s13054-020-02986-4)
Supplement: Supplementary file 5 — Additional file 5 : Table S5. Multivariable analysis (excluding 116 patients with negative cumulative fluid balance of day of initiation of CRRT). [file 13054_2020_2986_MOESM5_ESM.docx]

**Supplementary Table S5: Multivariable analysis (excluding 116 patients with negative cumulative fluid balance of day of initiation of CRRT)**

|  | **ICU mortality** | | | **Hospital mortality** | | |
| --- | --- | --- | --- | --- | --- | --- |
|  | **OR** | **95% CI** | **p-value** | **OR** | **95% CI** | **p-value** |
| **Age** | 1.02 | 1.01 - 1.04 | <0.001 | 1.02 | 1.01 - 1.03 | 0.003 |
| **Male sex** | 1.26 | 0.86 - 1.84 | 0.24 | 1.37 | 0.96 - 1.96 | 0.09 |
| **BMI**  **<20**  **20 to <25**  **25 to <30**  **30 to <40**  **40** | 2.48  1  0.86  0.85  0.62 | 1.00 – 6.13  0.54 – 1.35  0.53 – 1.38  0.27 -1.03 | 0.14 | 3.56  1  0.88  0.83  1.38 | 1.41 – 8.53  0.57 – 1.36  0.53 – 1.31  0.66 – 2.87 | 0.032 |
| **SOFA score on 1^st^ day of CRRT** | 1.18 | 1.12 - 1.25 | <0.001 | 1.14 | 1.08 - 1.20 | <0.001 |
| **Highest arterial lactate concentration on 1^st^ day of CRRT** (µmol/L) | 1.03 | 0.99 - 1.08 | 0.14 | 1.06 | 1.01 - 1.10 | 0.001 |
| **Hb on 1^st^ day of CRRT** (per 10g/dL) | 0.99 | 0.89 - 1.10 | 0.85 | 0.97 | 0.88 - 1.07 | 0.57 |
| **Daily noradrenaline dose** [µg], mean *****  **0**  **1 – 4,999**  **5,000 – 9,999**  **10,000 – 49,999**  $\boldsymbol{\geq}$**50,000** | 1  0.85  0.94  2.41  2.63 | 0.46 – 1.57  0.45 – 1.95  1.44 – 4.02  1.28 – 5.43 | <0.001 | 1  1.09  1.18  2.01  1.96 | 0.63 – 1.88  0.61 – 2.28  1.23 – 3.27  0.96 – 4.02 | 0.017 |
| **Cumulative FB at CRRT initiation** (per 1000ml) | 1.01 | 0.96 - 1.05 | 0.77 | 1.02 | 0.979 - 1.06 | 0.46 |
| **Delta cumulative FB**  **No nadir reached**  **<2500 ml reduction**  **2500 – 4999 ml reduction**  $\boldsymbol{\geq}$**5000 ml reduction** | 1  0.85  0.57  0.54 | 0.48 – 1.53  0.35 – 0.93  0.30 – 0.99 | 0.046 | 1  0.76  0.65  0.44 | 0.44 – 1.31  0.41 – 1.04  0.25 – 0.78 | 0.032 |

Abbreviations: BW = body weight; BMI = body mass index; CI = confidence interval; FB = fluid balance; Hb = haemoglobin in [g/dL]; ICU = intensive care unit; IQR = interquartile range; OR = odds ratio; CRRT = continuous renal replacement therapy; SOFA = Sequential Organ Failure Assessment

There were no significant interactions between cumulative and delta fluid balance (ICU model: p=0.92, hospital model: p=0.79).

C-statistic: ICU model c=0.77, hospital model c=0.74, Hosmer Lemeshow goodness of fit: ICU model: p=0.19, hospital model: p=0.27
