# Supplementary material for: Fluid removal associates with better outcomes in critically ill patients receiving continuous renal replacement therapy: a cohort study
Source: Crit Care. 2020 Jun 1;24:279. doi: 10.1186/s13054-020-02986-4 (PMC7268712; doi:10.1186/s13054-020-02986-4)
Supplement: Supplementary file 6 — Additional file 6 : Table S6. Multivariable analysis based on SOFA score at CRRT initiation. [file 13054_2020_2986_MOESM6_ESM.docx]

**Supplementary Table S6 Multivariable analysis based on SOFA score at CRRT initiation**

|  | **SOFA ≤10 at initiation of CRRT** | | | **SOFA >10 at initiation of CRRT** | | |
| --- | --- | --- | --- | --- | --- | --- |
|  | **OR** | **95% CI** | **p-value** | **OR** | **95% CI** | **p-value** |
| **ICU mortality** | | | | | | |
| ***Unadjusted analysis*** |  |  |  |  |  |  |
| Cum FB on 1^st^ day of CRRT initiation (per 1000ml) | 1.10 | 1.04-1.18 | 0.002 | 1.01 | 0.95-1.06 | 0.84 |
| Delta cumulative FB  No nadir reached  <2500 ml reduction  2500 – 4999 ml reduction  $\geq$5000 ml reduction | 1  0.86  0.80  0.78 | 0.67-1.11  0.63-1.04  0.62-0.97 | 0.09 | 1  0.58  0.49  0.24 | 0.48-0.70  0.41-0.59  0.21-0.28 | <0.001 |
| ***Adjusted analysis*** |  |  |  |  |  |  |
| Cum FB on 1^st^ day of CRRT initiation (per 1000ml) | 1.08 | 1.00-1.16 | 0.06 | 0.96 | 0.91-1.02 | 0.21 |
| Delta cumulative FB  No nadir reached  <2500 ml reduction  2500 – 4999 ml reduction  $\geq$5000 ml reduction | 1  1.55  0.98  1.28 | 0.64-3.79  0.44-2.14  0.53-3.09 | 0.68 | 1  0.75  0.51  0.42 | 0.34-1.64  0.27-0.95  0.19-0.91 | 0.029 |
| **Hospital mortality** | | | | | | |
| ***Unadjusted analysis*** |  |  |  |  |  |  |
| Cum FB on 1^st^ day of CRRT initiation (per 1000ml) | 1.05 | 0.99-1.11 | 0.08 | 1.03 | 0.97-1.08 | 0.33 |
| Delta cumulative FB  No nadir reached  <2500 ml reduction  2500 – 4999 ml reduction  $\geq$5000 ml reduction | 1  1.04  0.18  0.60 | 0.52-2.08  0.65-2.14  0.30-1.20 | 0.17 | 1  0.57  0.54  0.23 | 0.47-0.69  0.45-0.65  0.19-0.27 | <0.001 |
| ***Adjusted analysis*** |  |  |  |  |  |  |
| Cum FB on 1^st^ day of CRRT initiation (per 1000ml) | 1.06 | 0.99-1.13 | 0.10 | 0.99 | 0.93-1.04 | 0.67 |
| Delta cumulative FB  No nadir reached  <2500 ml reduction  2500 – 4999 ml reduction  $\geq$5000 ml reduction | 1  1.48  1.39  1.02 | 0.66-3.28  0.69-2.82  0.46-2.23 | 0.60 | 1  0.68  0.53  0.28 | 0.31-1.49  0.29-0.99  0.13-0.61 | 0.010 |

Abbreviations: Cum FB = cumulative FB; CRRT = continuous renal replacement therapy; SOFA = Sequential Organ Failure Assessment; CI = confidence interval; OR = odds ratio
